# Outcomes of Cardiac Resynchronization Therapy in Patients with Hypothyroidism and Heart Failure

**DOI:** 10.1186/s12872-020-01693-w

**Published:** 2020-09-23

**Authors:** Mei Yang, Xuping Li, John C. Morris, Jinjun Liang, Abhishek J. Deshmukh, David Hodge, Yigang Li, Yong-Mei Cha

**Affiliations:** 1 Department of Cardiology, Shanghai Jiao Tong University School of Medicine Xinhua Hospital, 1665 Kongjiang Road, Shanghai, China; 2 Department of Cardiovascular Diseases, Mayo Clinic, 200 First Street SW, Rochester, MN USA; 3 Department of Cardiovascular Medicine, The Second Xiangya Hospital, Central South University, 139 Renminzhong Road, Changsha, Hunan China; 4 Division of Endocrinology, Diabetes, Metabolism, and Nutrition, Mayo Clinic, 200 First Street SW, Rochester, MN 55905 USA; 5 Department of Cardiology, Renmin Hospital of Wuhan University, Hubei Zhang Road No. 99, Wuhan, Hubei China; 6 Department of Health Sciences Research, Mayo Clinic, 4500 San Pablo Road, Jacksonville, FL USA

## Abstract

**Background:**

Hypothyroidism is known to be associated with adverse clinical outcomes in heart failure. The association between hypothyroidism and cardiac resynchronization therapy outcomes in patients with severe heart failure is not clear.

**Methods:**

The study included 1316 patients who received cardiac resynchronization therapy between 2002 and 2015. Baseline demographics and cardiac resynchronization therapy outcomes, including left ventricular ejection fraction, New York Heart Association class, appropriate implantable cardioverter-defibrillator therapy, and all-cause mortality, were collected from the electronic health record.

**Results:**

Of the study cohort, 350 patients (26.6%) were classified as the hypothyroidism group. The median duration of follow-up was 3.6 years (interquartile range, 1.7–6.2 years). Hypothyroidism was not associated with a higher risk of all-cause mortality in patients receiving CRT for heart failure. The risk of appropriate implantable cardioverter-defibrillator therapy significantly increased in association with increased baseline thyroid-stimulating hormone level in the entire cohort (hazard ratio, 1.23 per 5mIU/L increase; 95% CI, 1.01–1.5; *P* = 0.04) as well as in the hypothyroid group (hazard ratio, 1.44 per 5mIU/L increase; 95% CI, 1.13–1.84; *P* = 0.004).

**Conclusions:**

CRT improves cardiac function in hypothyroid patients. The ventricular arrhythmic events requiring ICD therapies are associated with baseline TSH level, which might be considered as an important biomarker to stratify the risk of sudden death for patients with heart failure and hypothyroidism.

## Background

Thyroid hormones regulate cardiac contractility, relaxation and coronary blood flow. In patients with heart failure (HF), decreased thyroid function is associated with an increased risk of hospitalization, adverse cardiovascular outcomes, and mortality [1, 2]. Previous studies have shown the beneficial effects of synthetic thyroid hormone replacement therapy for improving left ventricular function in patients with HF [3, 4]. Multiple clinical trials have proven the efficacy of cardiac resynchronization therapy (CRT) in patients with severe left ventricular systolic dysfunction, mild to severe HF symptoms, and wide QRS complex [5, 6]. CRT improves free triiodothyronine levels and the free triiodothyronine/free thyrokine ratio, an effect that may be important in reversal of cardiac remodeling [7]. Despite the high incidence of hypothyroidism among CRT recipients, little is known about the outcomes of CRT in patients with HF and hypothyroidism. Therefore, the objective of this study was to determine the effects of hypothyroidism on clinical outcomes after CRT.

## Method

### Study patients

This retrospective cohort study included patients who received de novo CRT–defibrillator (CRT-D) or CRT–pacemaker (CRT-P) at Mayo Clinic between January 2002 and 2015. Indications for CRT were HF symptoms despite optimal medical therapy, left ventricular ejection fraction (LVEF) 35% or less, QRS duration 120 milliseconds or greater, and NYHA class III-IV (ACCF/HRS/AHA guideline [8] or NYHA class I-IV [9]. The following patients were excluded: 1) those who had device upgrade from CRT-P or CRT-D; 2) those with hyperthyroidism or a baseline thyroid-stimulating hormone (TSH) value less than 0.3 mIU/L; and 3) those who did not agree to the use of their records for research. The study was approved by the Mayo Clinic Institutional Review Board.

### Baseline evaluation and data collection

All patients were evaluated before CRT. LVEF was assessed with transthoracic electrocardiography. New York Heart Association (NYHA) functional class, the cause of HF, concomitant cardiovascular diseases, and echocardiographic variables were collected from the electronic health record. Information regarding medications, including thyroid replacement therapy (TRT), TSH values, and history of thyroid function was also assessed. All data were collected by one cardiologist blinded to the clinical outcome data. Baseline TSH levels were measured within 3 months before CRT. Data on appropriate implantable cardioverter-defibrillator (ICD) therapy were collected from the institutional ICD database and device interrogation reports. Survival data were collected from the electronic health records and the national death and location database (Accurint, Lexisnexis for Mayo Clinic patients).

### CRT implantation and follow-up

US Food and Drug Adminstration–approved, commercially available CRT-Ds or CRT-Ps were implanted in the pacing suite of Mayo Clinic, Rochester, Minnesota, between 2002 and 2015. The right ventricular lead and right atrial lead were positioned conventionally in the right ventricular apex and right atrial appendage, respectively. The left ventricular lead was placed preferentially in the posterolateral or lateral vein tributary via the coronary sinus. Patients were monitored overnight. The devices were interrogated, the device pockets were examined, and chest radiography was performed before the dismissal.

Patients were followed at 12-month after CRT. Response to CRT was defined as LVEF improvement of more than 5% at the echocardiogram follow-up. All patients were followed via remote monitoring system and had a device follow-up report every 3 months. On the CRT-D devices, tachycardia detection and therapy were programmed on a standard protocol for primary prevention of sudden death. The antitachycardia pacing was programmed as an initial therapy followed by high-energy shocks. Appropriate ICD therapy was defined as antitachycardia pacing or shock delivery for sustained ventricular fibrillation or ventricular tachycardia. Appropriateness of ICD therapies was adjudicated by an electrophysiologist or a specially trained device nurse on the basis of the stored intracardiac electrogram.

### Definition of hypothyroidism and euthyroidism

Hypothyroidism was defined as a documented diagnosis of hypothyroidism or subclinical hypothyroidism (SH) in the medical record or a TSH value of 5.0 mIU/L of more before CRT. The cutoff value of 5.0 mIU/L was determined by the upper limit of TSH reference range. Euthyroidism was defined as no documented history of hypothyroidism or hyperthyroidism in the medical record and a TSH value of 0.3 mIU/L to 5.0 mIU/L before CRT. Hypothyroidism was further divided into 2 categories: overt hypothyroidism (OH) and SH. OH was defined as a documented diagnosis of hypothyroidism or a baseline TSH value of 20 mIU/L or more, and SH was defined as a documented diagnosis of subclinical hypothyroidism or baseline TSH value of 5 mIU/L to 20 mIU/L without a documented diagnosis of thyroid dysfunction according to previously described TSH cutoffs and expert reviews [10, 11].

### Statistical analysis

Continuous variables are expressed as mean ± SD or median (25th, 75th percentile). The group comparison was performed with Student *t* test or the Wilcoxon rank-sum test, as appropriate. Categorical variables are presented as counts and percentages and were compared across groups using the χ2 test or Fisher exact test, as appropriate. Kaplan–Meier survival curves were generated, and the log-rank test was used to assess for differences between the curves. Univariate and multivariate Cox proportional hazards regression were used to determine the relationship between thyroid-related variables and outcomes. The start time was defined as the date of CRT implantation. The end time for survival analysis was defined as the date of death or the last follow-up date if the patient was alive. The end time for ICD therapy analysis was defined as the date of first appropriate ICD therapy or the last date of device interrogation if the patient had not received appropriate ICD detection and therapy. Only one thyroid-related variable was included in each multivariate model. All variables in Table 1 as well as CRT response were included in univariate analysis for prediction of outcomes. Only those predictors with *P* < 0.1 in univariate analysis were further included in the multivariate model as covariates. All *P* values were 2-sided, and a *P* value < 0.05 was considered to indicate statistical significance. All statistical analyses were performed using JMP Pro version 10.0.0 (SAS Institute, Inc).

**Table 1:** Baseline Characteristics

Variable	All (***n*** = 1316)	Euthyroidism (***n*** = 966)	Hypothyroidism (***n*** = 350)	***P*** Value
Age, y	69.8 ± 12.2	69.3 ± 12.4	71.2 ± 11.2	0.01
Female	289 (22.0)	184 (19.0)	105 (30.0)	< 0.001
CRT-D	1181 (89.7)	867 (89.7)	314 (90.0)	0.87
Primary prevention	979 (82.9)	749 (86.5)	230 (73.0)	< 0.001
Hypertension	539 (41.0)	398 (41.2)	141 (40.4)	0.81
Diabetes mellitus	398 (30.2)	297 (30.7)	101 (28.9)	0.54
Chronic kidney disease	357 (27.1)	248 (25.7)	109 (31.2)	0.04
Hyperlipidemia	600 (45.7)	442 (45.7)	158 (45.4)	0.92
Atrial fibrillation history	599 (44.5)	430 (44.5)	169 (48.4)	0.20
ICM	632 (48.0)	462 (47.8)	170 (48.7)	0.77
LBBB	644 (40.1)	491 (52.2)	153 (44.5)	0.02
QRS duration, ms	170.2 ± 28.5	169.5 ± 28.2	172.1 ± 29.4	0.16
NYHA functional class, 1–4	2.8 ± 0.6	2.8 ± 0.6	2.9 ± 0.5	0.64
3–4	892	644 (69.7)	248 (73.6)	
2	349	264 (28.6)	85 (25.2)	
1	20	16 (1.7)	4 (1.2)	
LVEF, %	24.3 ± 6.5	24.2 ± 6.6	24.4 ± 6.6	0.62
TRT	254 (19.3)	0 (0)	254 (72.9)	< 0.001
Amiodarone	232 (17.6)	135 (14.0)	97 (27.9)	< 0.001
Statin	705 (53.6)	529 (54.7)	176 (50.4)	0.17
Digoxin	539 (41.0)	391 (40.4)	148 (42.4)	0.52
β-blocker	1156 (87.8)	853 (88.2)	303 (86.8)	0.50
ACEI/ARB	1054 (80.1)	781 (80.8)	273 (78.2)	0.31
Aldactone	391 (29.7)	288 (29.8)	103 (29.5)	0.93
Furosemide	886 (67.3)	640 (66.2)	246 (70.5)	0.14

Values are count (%) or mean ± SD

*Abbreviations*: *ACEI* angiotensin-converting enzyme inhibitor, *ARB* angiotensin receptor blocker, *CRT-D* cardiac resynchronization therapy-defibrillator, *ICM* ischemic cardiomyopathy, *LBBB* left bundle branch block, *LVEF* left ventricular ejection fraction, *NYHA* New York Heart Association, *TRT* thyroid replacement therapy

## Results

### Baseline characteristics

Of 1316 patients included in the study, 1181 (89.7%) patients received CRT-D, among whom 979 (82.9%) CRT-Ds were implanted for primary prevention. Of all the patients, 350 (26.6%) were categorized as the hypothyroidism group: 260 had a known diagnosis of hypothyroidism, and 90 had the diagnosis on the basis of their increased TSH value. In the hypothyroidism group, 85 patients (24.3%) were categorized as having SH and 265 (75.7%) as having OH. The baseline patient characteristics are presented in Table 1. Compared with the euthyroid group, patients with hypothyroidism were significantly older and were more likely to be female, to have chronic kidney disease, and to use amiodarone. There were 254 patients (72.9%) in the hypothyroidism group but none in the SH group were receiving TRT. The median baseline TSH value was 2.6 mIU/L (interquartile range, 1.6–4). Patients with hypothyroidism had significantly higher TSH values than those with euthyroidism (5.7 ± 5.7mIU/L vs. 2.4 ± 1.1mIU/L, *P* < 0.001).

### Improvement in HF after CRT

Of 1316 study patients, 826 (62.8%) returned for an in-clinic follow-up in 12-month to assess CRT response. The NYHA class improvement in the hypothyroidism group was significantly less than that in the euthyroid group (− 0.4 ± 0.8 vs. -0.6 ± 0.8, *P* = 0.002). The LVEF improvement was similar in the hypothyroid and the euthyroid groups (8.3% ± 11.8% vs. 9.3% ± 12.3%, *P* = 0.32). The CRT response rate was 51.5% in the hypothyroid group and 54.1% in the euthyroid group (*P* = 0.51). For patients with LBBB, the response rate in hypothyroid and euthyroid group was 62.5 and 57.8% respectively (*P* = 0.46). The NYHA improvement was similar in 2 groups (− 0.6 ± 0.7 vs. -0.7 ± 0.8, *P* = 0.26). So was the LVEF improvement (11.9% ± 13.6% vs. 11.3% ± 13.1%, *P* = 0.72).

### All-cause mortality

**Fig. 1:**
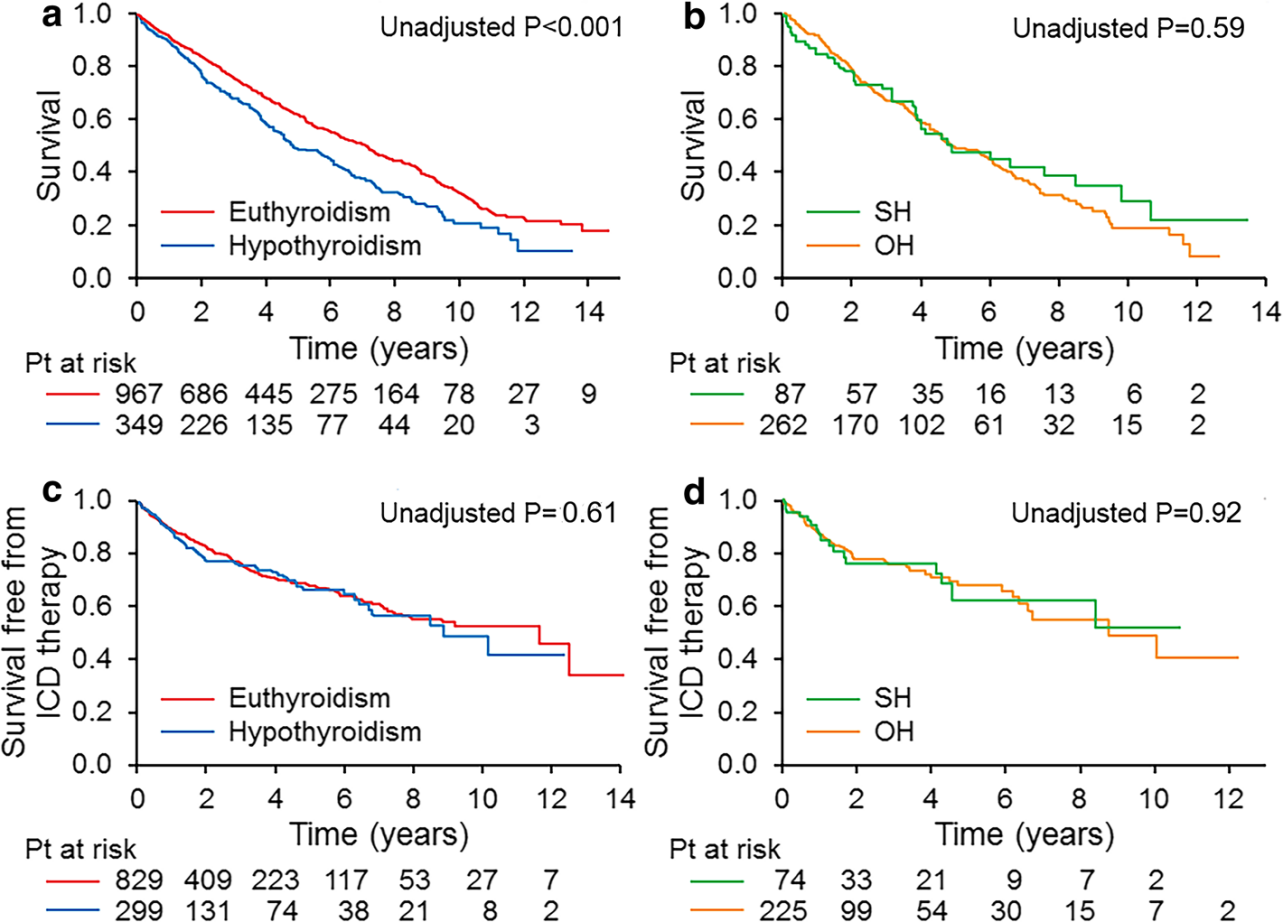
Kaplan–Meier survival comparison in patients with hypothyroidism and euthyroidism (**a**), and in patients with subclinical (SH) and overt hypothyroidism (OH) (**b**); Kaplan–Meier survival free from appropriate implantable cardioverter-defibrillatory (ICD) therapy in patients with hypothyroidism and euthyroidism (**c**) and in patients with SH and OH (**d**)

The median duration from the date of CRT implant to the last follow-up date or the date of death was 3.6 years (interquartile range, 1.7–6.2 years). During the follow up, 618 patients died. The mean survival duration for these patients was 3.7 ± 3.0 years. Kaplan–Meier estimates showed a significantly higher all-cause mortality in the hypothyroid group (78.2%) than in the euthyroidism group (67.0%) at 10-year follow-up (*P* < 0.001) (Fig. 1a). After adjustment for covariates, neither hypothyroidism nor baseline TSH was an independent predictor for all-cause mortality (Table 2).

**Table 2:** Univariate and Multivariate Cox Regression Models to Identify Predictors for All-Cause Mortality in All Patients

Variable	Univariate Analysis			Multivariate Analysis^a^			Multivariate Analysis^b^		
	HR	95% CI	***P*** Value	HR	95% CI	***P*** Value	HR	95% CI	***P*** Value
Age	1.04	1.04–1.05	< 0.001	1.04	1.03–1.06	< 0.001	1.04	1.03–1.06	< 0.001
Female	0.54	0.44–0.68	< 0.001	0.73	0.52–1.04	0.09	0.77	0.53–1.12	0.18
CRT-D	0.71	0.56–0.91	0.007	0.72	0.47–1.12	0.14	0.74	0.45–1.22	0.23
Hypertension	0.97	0.83–1.14	0.73						
Diabetes mellitus	1.49	1.26–1.76	< 0.001	1.77	1.35–2.31	< 0.001	1.84	1.35–2.49	< 0.001
Chronic kidney disease	2.46	2.09–2.89	< 0.001	1.47	1.12–1.93	0.006	1.45	1.07–1.98	0.02
Hyperlipidemia	0.99	0.85–1.16	0.94						
Atrial fibrillation	1.33	1.13–1.55	< 0.001	1.18	0.91–1.53	0.20	1.07	0.79–1.43	0.67
ICM	2.02	1.71–2.38	< 0.001	1.14	0.86–1.50	0.36	1.15	0.856–1.57	0.37
LBBB	0.70	0.60–0.83	< 0.001	0.75	0.58–0.97	0.03	0.78	0.58–1.05	0.11
QRS duration	1.00	0.99–1.00	0.63						
NYHA functional class	1.95	1.66–2.30	< 0.001	1.30	1.01–1.68	0.05	1.24	0.93–1.65	0.14
LVEF	0.99	0.98–1.00	0.064	0.98	0.96–1.00	0.03	0.98	0.96–1.00	0.10
TRT	1.38	1.14–1.67	0.001	1.20	0.71–2.04	0.50	1.43	1.02–2.02	0.04
Amiodarone	1.33	1.09–1.61	0.005	1.20	0.87–1.66	0.27	1.25	0.89–1.76	0.20
Statin	1.05	0.90–1.23	0.54						
Digoxin	1.31	1.12–1.54	0.001	1.42	1.10–1.85	0.008	1.46	1.09–1.95	0.01
b-Blocker	0.90	0.71–1.15	0.41						
ACEI/ARB	0.67	0.55–0.81	< 0.001	0.86	0.61–1.23	0.41	0.76	0.52–1.11	0.16
Aldactone	1.00	0.84–1.18	0.96						
Furosemide	1.36	1.14–1.64	0.001	1.26	0.93–1.71	0.14	1.3	0.91–1.86	0.15
CRT response	0.41	0.32–0.52	< 0.001	0.42	0.32–0.54	< 0.001	0.45	0.33–0.60	< 0.001
Hypothyroidism	1.37	1.15–1.62	< 0.001	1.16	0.72–1.89	0.54	–	–	–
TSH per 5 mIU/L	1.41	1.29–1.55	< 0.001	–	–	–	0.94	0.74–1.19	0.60

^a^ Multivariate analysis including Hypothyroidism. ^b^ Multivariate analysis including TSH value. Abbreviations as in Table 1. *TSH* thyroid-stimulating hormone

In the hypothyroid group, although the 10 year mortality rates were lower for SH than for OH (70.2% vs. 80.4%), there was no statistical difference between the Kaplan-Meier curves (Fig. 1b, *P* = 0.59). Baseline TSH value was not associated with all-cause mortality (*P* = 0.12).

### Appropriate ICD therapy

In 1181 patients who received CRT-D, 270 (22.9%) had a ventricular tachycardia or ventricular fibrillation event treated with ICD therapy. Of these patients, 128 (47.4%) had successful treatment with initial ATP, and 142 (52.6%) received shock therapy initially or after failed antitachycardia pacing. The median time from CRT-D implantation to the first therapy was 16.2 months (interquartile range, 5.6–35.6 months). The event rate of appropriate ICD therapy was similar between the hypothyroidism and euthyroidism groups (Fig. 1c). Hypothyroidism was not associated with higher risk of appropriate ICD therapy (*P* = 0.61). Baseline TSH value was independently associated with appropriate ICD therapy (hazard ratio, 1.23 per 5mIU/L increase; 95% CI, 1.01–1.5; *P* = 0.04, Table 3).

**Table 3:** Univariate and Multivariate Cox Regression Models to Identify Predictors for appropriate ICD therapy in All Patients

Variable	Univariate Analysis			Multivariate Analysis		
	HR	95% CI	***P*** Value	HR	95% CI	***P*** Value
Age	0.99	0.98–1.00	0.08			
Female	0.41	0.29–0.59	< 0.001	0.45	0.27–0.75	0.002
Primary prevention	0.54	0.40–0.72	< 0.001	0.28	0.10–0.83	0.02
Hypertension	1.04	0.81–1.32	0.78			
Diabetes mellitus	1.01	0.78–1.32	0.92			
Chronic kidney disease	1.24	0.94–1.63	0.13			
Hyperlipidemia	1.09	0.86–1.39	0.48			
Atrial fibrillation	1.20	0.94–1.53	0.14			
ICM	1.13	0.89–1.44	0.32			
LBBB	0.67	0.52–0.85	0.001	0.72	0.50–1.05	0.08
QRS duration	0.997	0.992–1.001	0.17			
NYHA functional class	1.16	0.91–1.47	0.24			
LVEF	0.98	0.96–1.00	0.02	0.97	0.95–1.01	0.11
TRT	0.97	0.71–1.32	0.84			
Statin	1.02	0.80–1.30	0.87			
amiodarone	1.62	1.22–2.15	0.001	0.44	0.16–1.27	0.13
Digoxin	1.55	1.22–1.97	< 0.001	1.44	1.00–2.07	0.05
ACEARB	0.77	0.57–1.05	0.09	0.73	0.45–1.18	0.20
Aldactone	1.07	0.82–1.38	0.62			
Furosemide	1.30	0.99–1.71	0.06	1.13	0.75–1.70	0.54
CRT response	0.43	0.32–0.59	< 0.001	0.46	0.32–0.68	< 0.001
TSH per 5 mIU/L	1.05	1.02–1.09	0.002	1.23	1.01–1.50	0.04

Abbreviations as in Table 1. *TSH* thyroid-stimulating hormone

In the hypothyroid group, the event rate of appropriate ICD therapy was similar between the SH and OH groups (Fig. 1d). However, the baseline TSH value was an independent predictor for appropriate ICD therapy (hazard ratio, 1.44 per 5mIU/L increase; 95% CI, 1.13–1.84; *P* = 0.004, Table 4).

**Table 4:** Univariate and Multivariate Cox Regression Models to Identify Predictors for Appropriate ICD Therapy in Patients with Hypothyroidism

Variable	Univariate Analysis			Multivariate Analysis		
	HR	95% CI	***P*** Value	HR	95% CI	***P*** Value
Age	0.99	0.97–1.01	0.42			
Female	0.33	0.17–0.64	0.001	1.59	0.66–3.86	0.30
Primary prevention	0.46	0.28–0.74	0.002	0.15	0.02–1.21	0.07
Hypertension	0.85	0.53–1.38	0.52			
Diabetes mellitus	0.79	0.45–1.38	0.41			
Chronic kidney disease	1.90	1.16–3.11	0.01	1.81	0.95–3.48	0.07
Hyperlipidemia	0.75	0.47–1.22	0.25			
Atrial fibrillation	1.18	0.74–1.89	0.48			
ICM	1.60	0.98–2.59	0.06	0.99	0.51–1.95	0.98
LBBB	0.74	0.46–1.19	0.22			
QRS duration	1.00	0.99–1.01	0.78			
NYHA functional class	1.31	0.80–2.17	0.29			
LVEF	0.99	0.95–1.03	0.55			
TRT	0.74	0.45–1.23	0.25			
amiodarone	1.79	1.10–2.89	0.02	0.36	0.05–2.81	0.33
Statin	1.14	0.71–1.83	0.57			
Digoxin	1.03	0.64–1.65	0.91			
b-Blocker	0.87	0.45–1.65	0.66			
ACEI/ARB	0.78	0.45–1.36	0.39			
Aldactone	0.68	0.39–1.17	0.16			
Furosemide	1.23	0.71–2.11	0.46			
CRT Response	0.44	0.24–0.80	0.007	0.58	0.31–1.12	0.11
TSH per 5 mIU/L	1.35	1.12–1.63	0.002	1.44	1.13–1.84	0.004

Abbreviations as in Table 1. *TSH* thyroid-stimulating hormone

### Amiodarone

**Fig. 2:**
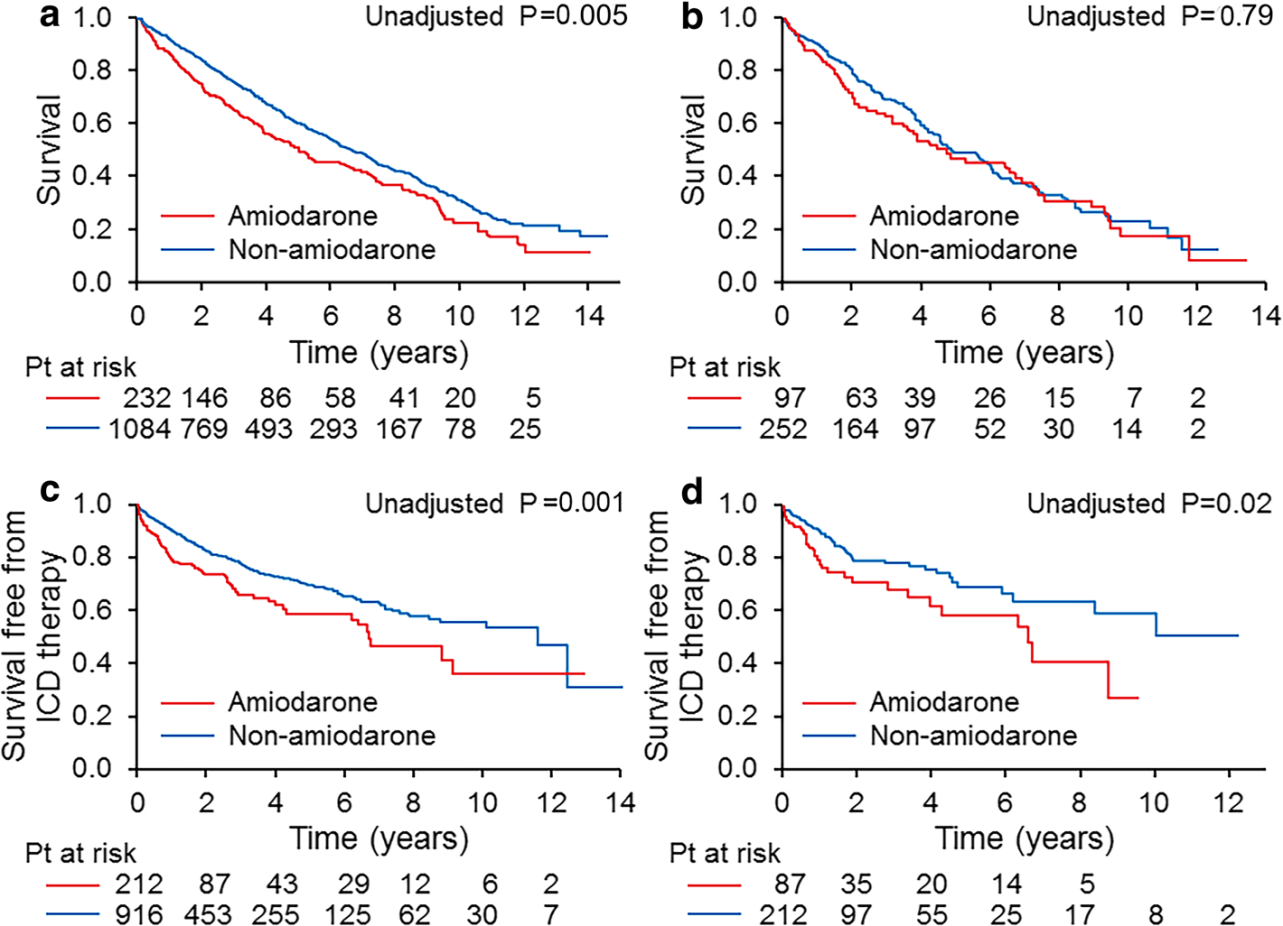
Kaplan–Meier survival according to amiodarone usage in entire cohort (**a**) and in patients with hypothyroidism (**b**), Kaplan–Meier survival free from appropriate implantable cardioverter-defibrillator (ICD) therapy according to amiodarone usage in entire cohort (**c**) and in patients with hypothyroidism (**d**)

In this study, amiodarone was prescribed in 232 patients, 123 for ventricular tachycardia, 11 for atrial fibrillation and 98 for both ventricular tachycardia and atrial fibrillation. Amiodarone was prescribed in 135 patients (14.0%) in the euthyroidism group and in 97 (27.9%) in the hypothyroidism group (*P* < 0.001). Kaplan–Meier estimates showed a higher risk of all-cause mortality and appropriate ICD therapy in patients receiving amiodarone than in those who were not in the entire cohort (both *P* **<** 0.05). In the hypothyroidism group, of the 97 patients with hypothyroidism who were prescribed amiodarone, 65 (67.0%) were receiving TRT. Patients who were receiving amiodarone had a higher risk of appropriate ICD therapy than those who were not receiving it (*P* = 0.02, Fig. 2). However, amiodarone was not an independent predictor for either all-cause mortality or appropriate ICD therapy (Tables 2, 3 and 4).

## Discussion

Our study had 2 main findings: 1) hypothyroidism is not associated with a higher risk of all-cause mortality in patients receiving CRT for HF, and 2) baseline TSH level is an independent predictor for appropriate ICD therapy. This is the first study to show that in patients who have HF, TSH level is associated with ventricular arrhythmic events after CRT.

### Hypothyroidism and CRT outcomes

The prevalence of hypothyroidism in patients with HF is 4 to 24% [2]. Our study showed a higher incidence of hypothyroidism than previous reports. Hypothyroidism is associated with adverse effects on cardiac output, cardiac contractility, and vascular resistance [12]. A decrease in the thyroid hormone level may result in endothelial dysfunction, vascular smooth muscle cell apoptosis, atherosclerosis or ischemic heart disease, and cardiac atrophy with chamber dilatation by sarcomere lengthening [13–15]. One study showed that a decrease in the thyroid hormone level is proportional to the severity of HF symptoms [16]. These pathophysiologic factors may diminish an optimal response to CRT. In this study, the hypothyroidism group had less improvement in NYHA class than the euthyroidism group, yet the improvement in LVEF was similar between the two groups at 1-year follow-up.

Hypothyroidism is associated with a higher mortality rate, cardiovascular events, and progression of chronic kidney disease and diabetes [1, 2, 10, 17–19]. Similarly, Chen’s study found that a low-normal free triiodothyronine level was associated with HF rehospitalization and overall mortality in euthyroid patients with HF receiving CRT [20]. In contrast, similar risk of all-cause mortality was found in our patients with hypothyroidism or euthyordism. This might be explained by the similar CRT response rate between them, implying the effectiveness and importance of CRT for patients with HF and hypothyroidism.

### Subclinical hypothyroidism

OH is associated with increased cardiovascular risks, whereas the risk with SH is still controversial [10, 21]. There is no consensus on whether SH warrants treatment [22]. One important reason for the controversy is that observational studies show that TSH tends to increase with age [23]. While age-based cut-off points for hypothyroidism not yet been standardized, there might be a danger of overtreatment, especially in the elderly. A large observational study has corroborated that TRT may minimize the risk of coronary heart disease in younger patients (< 70 years) [24]. A recent randomized, double-blind, placebo-controlled clinical trial found no difference in systolic or diastolic heart function after TRT compared with placebo in older adults (≥65 years) with subclinical hypothyroidism [25]. No comparison about TRT for SH patients was conducted in this study since no TRT was used for SH. Nevertheless, no differences were found in the outcomes between patients with SH and those with OH. Numerous studies have reported improvement in vascular and cardiac function and arterial stiffness in patients with SH [26, 27]. A meta-analysis showed a possible relationship between SH and an increased risk of coronary heart disease mortality compared with euthyroidism [28]. TRT was observed beneficial in preventing major adverse cardiovascular and cerebral events for hypothyroidism patients undergoing percutaneous coronary intervention [15]. Therefore, TRT should be individualized for SH patients, and more considered especially for those with high coronary heart diseases and younger age.

### Hypothyroidism and ventricular arrhythmia

Hypothyroidism is associated with both supraventricular and ventricular arrhythmias [29, 30]. QT interval prolongation and dispersion have been observed in patients with decreased thyroid function, which might be responsible for the increased inhomogeneity of ventricular repolarization and myocardial vulnerability to ventricular arrhythmia [31, 32]. These changes can be reversed after levothyroxine treatment [33, 34]. Not surprisingly, our patients with more severe thyroid dysfunction (higher TSH level) had higher risk of ventricular arrhythmic events despite CRT, an indication that ventricular arrhythmia is substantially associated with systemic diseases and comorbidities in HF, especially thyroid dysfunction. Indeed, our study is the first to demonstrate an increased baseline TSH level is associated with increased ventricular tachyarrhythmic events. Furthermore, the TSH level was an independent predictor for sustained ventricular arrhythmia. The TSH level might be considered as an important biomarker to stratify the risk of sudden death. Female sex was an independent predictor for fewer appropriate ICD therapies in our study, consistent with a previous report [35].

### Amiodarone and hypothyroidism

Amiodarone therapy frequently causes thyroid dysfunction, most commonly hypothyroidism. This effect is largely related to the high iodine content of the medication and its effect on thyroid hormone synthesis and release. Patients who receive CRT for HF more often receive amiodarone therapy for control of ventricular or atrial arrhythmia, in whom hypothyroidism may occur as an adverse drug effect. As Amiodarone is the most effective drug in controlling ventricular arrhythmia, TRT is accepted to overcome the adverse effect. In this study abnormal TSH level were associated with worse prognosis regardless of amiodarone usage or TRT. Moreover, the survival rate was lower in the amiodarone group than in the non-amiodarone group. However, amiodarone was not an independent predictor for survival, suggesting the amiodarone group was more likely to be sicker, requiring antiarrhythmic drug to control ventricular or atrial arrhythmia associated with heart failure. Our finding was in agreement with the finding of previous study in which patients who took amiodarone for rhythm control of atrial fibrillation had a higher risk of cardiovascular hospitalization and mortality rates compared to the rate control group [36]. Despite amiodarone reducing ICD shocks according to previous study [37], in our study the amiodarone group had a higher appropriate ICD therapy event rate than the non-amiodarone group, a suggestion of incomplete control of ventricular arrhythmia in this patient group.

### Limitations

This was a single-center retrospective cohort study without a 10 year follow-up schedule and was subject to the inherent limitations of retrospective study, like the missing data regarding therapy or TSH level changes during follow-up. Because triiodothyronine and thyroxine levels were not available, the thyroid status was assessed only by documented history and TSH levels. The TSH was measured only once before CRT implantation, the diagnosis of subclinical hypothyroidism may not be accurate. Finally, the national death and location database was used for survival analysis, with the limitation of knowing the cause of deaths.

## Conclusion

CRT improves cardiac function in hypothyroid patients. The ventricular arrhythmic events requiring ICD therapies are associated with baseline TSH level, which might be considered as an important biomarker to stratify the risk of sudden death for patients with heart failure and hypothyroidism.

### Ethics approval and consent for publication

The study was approved by the Mayo Clinic Institutional Review Board. The participants gave their written informed consent.

## Data Availability

The datasets used and/or analysed during the current study available from the corresponding author on reasonable request.

## Abbreviations

HF: Heart failure
CRT: Cardiac resynchronization therapy
CRT-D: CRT–defibrillator
CRT-P: CRT–pacemaker
LVEF: Left ventricular ejection fraction
TSH: Thyroid-stimulating hormone
NYHA: New York heart association
TRT: Thyroid replacement therapy
ICD: Implantable cardioverter-defibrillator
SH: Subclinical hypothyroidism
OH: Overt hypothyroidism
